# Supplementary material for: Determining the Authenticity of Spirulina Dietary Supplements Based on Stable Isotope and Elemental Composition
Source: Foods. 2023 Jan 27;12(3):562. doi: 10.3390/foods12030562 (PMC9914286; doi:10.3390/foods12030562)
Supplement: Supplementary file 1 [file foods-12-00562-s001.zip › foods-2165200-supplementary.pdf]

Table S1. Elemental composition of *Spirulina* dietary supplements from the Slovenian market.

S. No. [1]	Si (g/kg)	P (g/kg)	S (g/kg)	Cl (g/kg)	K (g/kg)	Ca (g/kg)	Ti (mg/kg)	Mn (mg/kg)	Fe (g/kg)	Zn (mg/kg)	Br (mg/kg)	Rb (mg/kg)	Sr (mg/kg)
S1	1.57	10.5	7.53	0.66	16.1	8.18	18.2	43.4	0.81	14.0	1.29	1.55	37.9
S2	7.69	11.4	7.53	3.07	14.8	1.32	56.9	159	3.29	43.6	11.2	7.47	10.0
S3	1.16	10.6	8.64	3.18	15.4	0.82	5.45	29.4	0.49	8.33	1.91	1.67	17.8
S4	13.5	10.0	7.57	5.77	17.1	2.20	46.5	128	3.48	52.7	16.5	11.9	24.3
S5	1.34	12.4	9.04	3.78	20.8	0.74	8.77	22.1	0.48	14.6	1.77	1.23	25.1
S6	21.7	12.7	6.47	0.09	11.0	1.28	9.31	54.9	0.90	13.9	0.50	4.12	10.2
S7	10.7	11.8	7.77	0.60	14.3	5.10	3.96	32.8	0.37	10.4	0.47	0.81	22.1
S8	16.0	11.5	7.53	1.03	14.7	2.04	60.6	35.0	1.39	16.0	0.91	0.91	27.3
S9	5.21	5.06	3.14	2.61	19.7	2.83	10.4	43.0	0.44	19.1	10.8	3.75	12.7
S10	2.34	14.1	9.38	2.11	18.5	3.09	35.5	36.6	1.68	18.5	1.34	1.58	34.7
S11	1.42	12.9	8.29	0.48	15.2	1.20	4.42	26.5	0.57	11.1	1.19	1.07	28.2
S12	1.63	13.9	8.42	1.77	16.8	5.24	12.3	38.2	1.38	33.0	1.39	1.48	31.8
S13	16.6	12.6	7.79	1.97	15.5	5.39	14.8	34.4	1.79	33.6	1.47	0.92	31.1
S14	7.94	12.2	7.54	0.19	13.6	1.00	9.91	34.9	0.69	16.5	0.57	2.23	7.41
S15	1.43	11.9	7.32	0.21	13.7	0.89	5.07	33.3	0.66	15.7	0.48	1.60	6.82
S16	1.59	14.7	7.50	0.52	14.3	2.78	6.08	36.3	0.65	17.5	0.91	0.50	12.7
S17	1.61	13.5	8.64	2.21	16.6	5.34	12.3	30.9	1.74	34.9	1.88	1.20	32.3
S18	2.74	12.6	6.17	0.60	8.63	63.5	43.1	47.1	0.75	11.1	9.11	2.55	478
S19	19.4	12.2	9.29	2.55	18.4	28.0	15.9	30.3	0.56	9.74	2.26	1.24	27.6
S20	16.8	12.1	8.39	3.04	16.3	2.43	47.5	29.1	1.13	23.0	2.00	1.91	32.2
S21	14.7	9.77	7.30	1.94	16.2	1.35	28.2	150	0.69	22.4	3.09	2.97	9.66
S22	1.85	6.82	3.60	0.55	7.40	1.37	11.1	21.1	0.39	7.69	1.24	1.06	8.00
S23	15.1	11.2	7.12	1.12	14.2	2.03	65.8	28.3	1.39	14.0	0.88	0.78	28.0
S24	1.40	9.27	6.05	0.87	12.5	2.45	19.0	88.3	0.77	15.4	1.04	1.16	11.2
S25	15.4	9.61	8.32	2.68	17.3	1.02	9.37	51.5	0.60	10.0	1.60	1.43	18.7
S26	15.0	10.9	7.91	5.63	17.5	2.28	42.3	185	3.09	35.5	17.4	9.96	14.1
S27	1.79	11.2	8.30	2.07	16.8	0.80	8.61	22.8	0.72	15.6	1.27	1.04	22.3
S28	4.91	10.3	8.66	3.47	17.4	0.72	2.58	26.2	0.47	7.27	2.54	2.44	15.4
S29	15.6	10.9	7.24	1.67	15.6	1.62	35.7	192	1.14	20.9	2.71	2.06	15.6
S30	15.1	10.1	6.99	1.63	15.2	1.50	34.5	195	1.12	21.7	3.21	1.33	15.2
S31	1.53	11.4	6.72	1.36	14.7	1.67	13.0	178	0.78	22.0	1.82	2.85	7.85
S32	16.1	10.2	8.15	2.62	14.2	2.54	39.4	35.9	1.39	22.9	1.86	2.30	35.5
S33	1.06	10.2	7.99	2.18	14.9	1.00	5.84	28.0	0.64	5.42	1.62	1.55	29.7
S34	16.9	10.9	8.09	4.34	15.9	0.91	7.82	27.8	0.52	11.3	4.47	0.57	31.2
S35	7.52	10.1	7.85	2.20	14.4	0.96	6.85	26.5	0.63	7.18	2.38	1.27	26.3
S36	1.97	11.4	7.10	2.58	14.9	2.17	24.7	34.5	1.01	17.0	6.74	1.23	55.0
S37	6.43	6.16	3.88	0.91	5.83	0.75	12.8	19.3	0.28	6.02	1.67	0.55	9.75
S38	7.56	10.3	8.04	0.30	9.00	4.93	2.81	24.9	0.41	13.3	0.67	1.33	26.1
S39	12.1	9.93	6.71	1.76	12.7	0.97	8.88	14.7	0.44	13.1	1.86	0.99	19.8
S40	8.49	10.2	8.06	0.34	8.84	5.52	4.39	28.3	0.42	14.7	0.85	1.09	29.3
S41	0.94	8.64	7.18	5.03	16.4	0.52	3.58	18.6	0.38	10.3	2.93	2.42	15.9
S42	1.07	10.1	7.72	4.70	15.1	0.69	8.89	29.1	0.55	9.91	3.78	1.21	23.7
S43	1.34	10.1	9.91	5.34	15.7	1.42	3.16	23.2	0.41	2.30	5.51	1.34	71.8
S44	0.78	8.56	6.63	2.75	20.6	3.45	10.4	84.9	0.69	24.9	8.00	6.55	86.2
S45	1.02	11.0	7.87	1.13	14.3	1.26	3.18	27.4	0.45	18.1	1.43	1.47	22.0
S46	0.68	6.64	7.38	5.36	26.9	0.46	5.36	32.9	0.93	7.59	7.07	4.21	4.39

[1] Sample number.

Table S2. Composition of Zarrouk's medium [1].

**Constituents**	Content (g/L)
K_2HPO_4	0.5
$NaNO_3$	2.5
K_2SO_4	1.0
NaCl	1.0
$MgSO_4 \cdot 7H_2O$	0.2
$CaCl_2 \cdot 2H_2O$	0.04
$FeSO_4 \cdot 7H_2O$	0.01
EDTA	0.08
$NaHCO_3$	16.8
	Content (mL)
Micronutrient solution (H_3BO_3 (2.86 g/L), $MnCl_2 \cdot 4H_2O$ (1.81 g/L), $ZnSO_4 \cdot 4H_2O$ (0.222 g/L), Na_2MoO_4 (0.0177 g/L), $CuSO_4 \cdot 5H_2O$ (0.079 g/L))	1.0

Reference:

1. Michael, A.; Kyewalyanga, M.S.; Mtolera, M.S.; Lugomela, C.V. Antioxidants Activity of the Cyanobacterium, *Arthrospira* (*Spirulina*) *Fusiformis* Cultivated in a Low-Cost Medium. *Afr. J. Food Sci.* **2018**, *12*, 188–195, doi:10.5897/AJFS2018.1688.

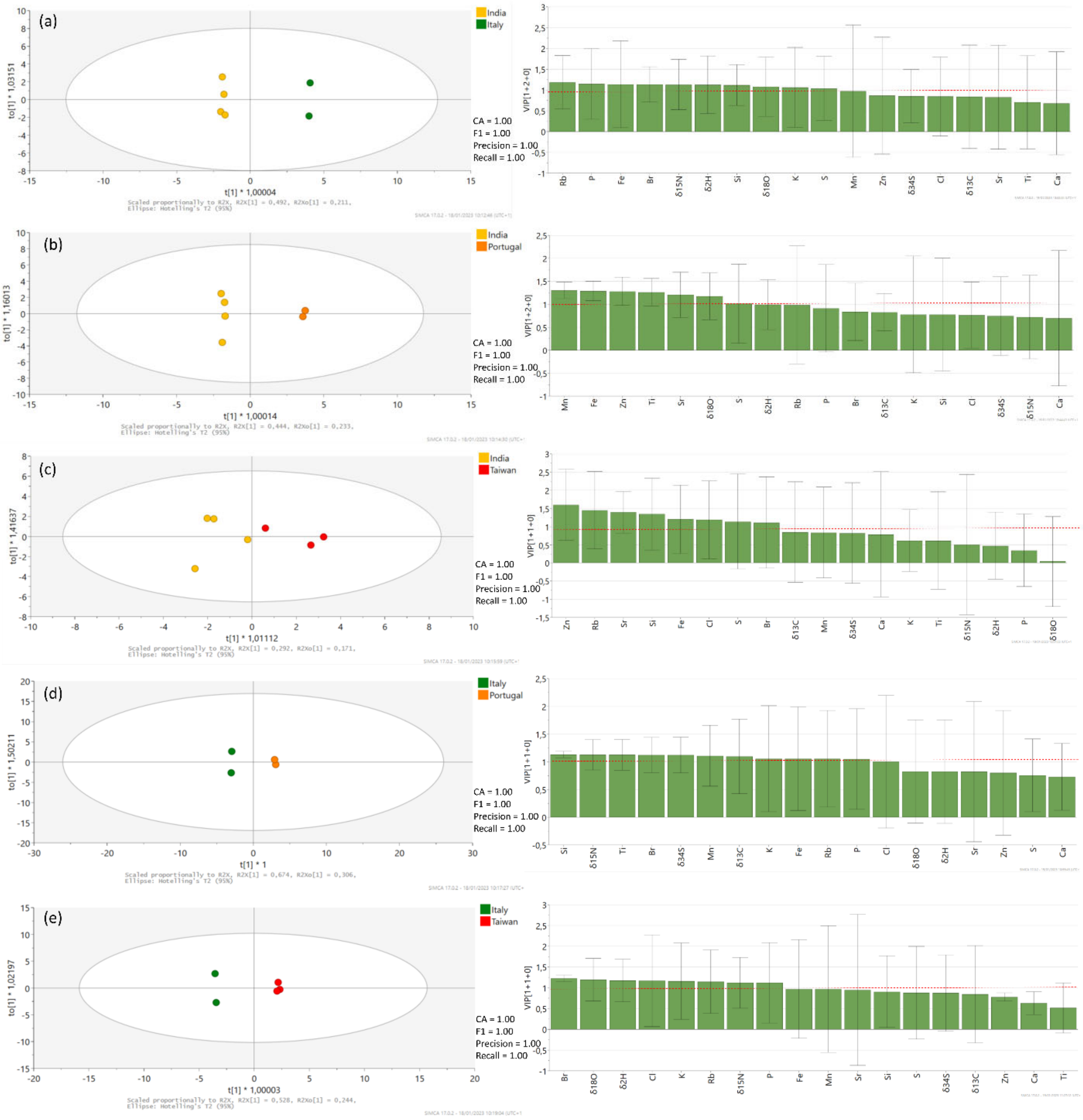

Figure S1: Pairwise comparisons between different declared countries of origin of *Spirulina* products: OPLS-DA score plots and VIP values, derived from all isotopic and elemental composition data. The ellipse on the score plot represents the 95% confidence interval. Red dotted line indicates a criteria for identification of the variables, important for the developed model. Separation of India from Italy (a), Portugal (b), Taiwan (c) and separation of Italy from Portugal (d) and Taiwan (e).

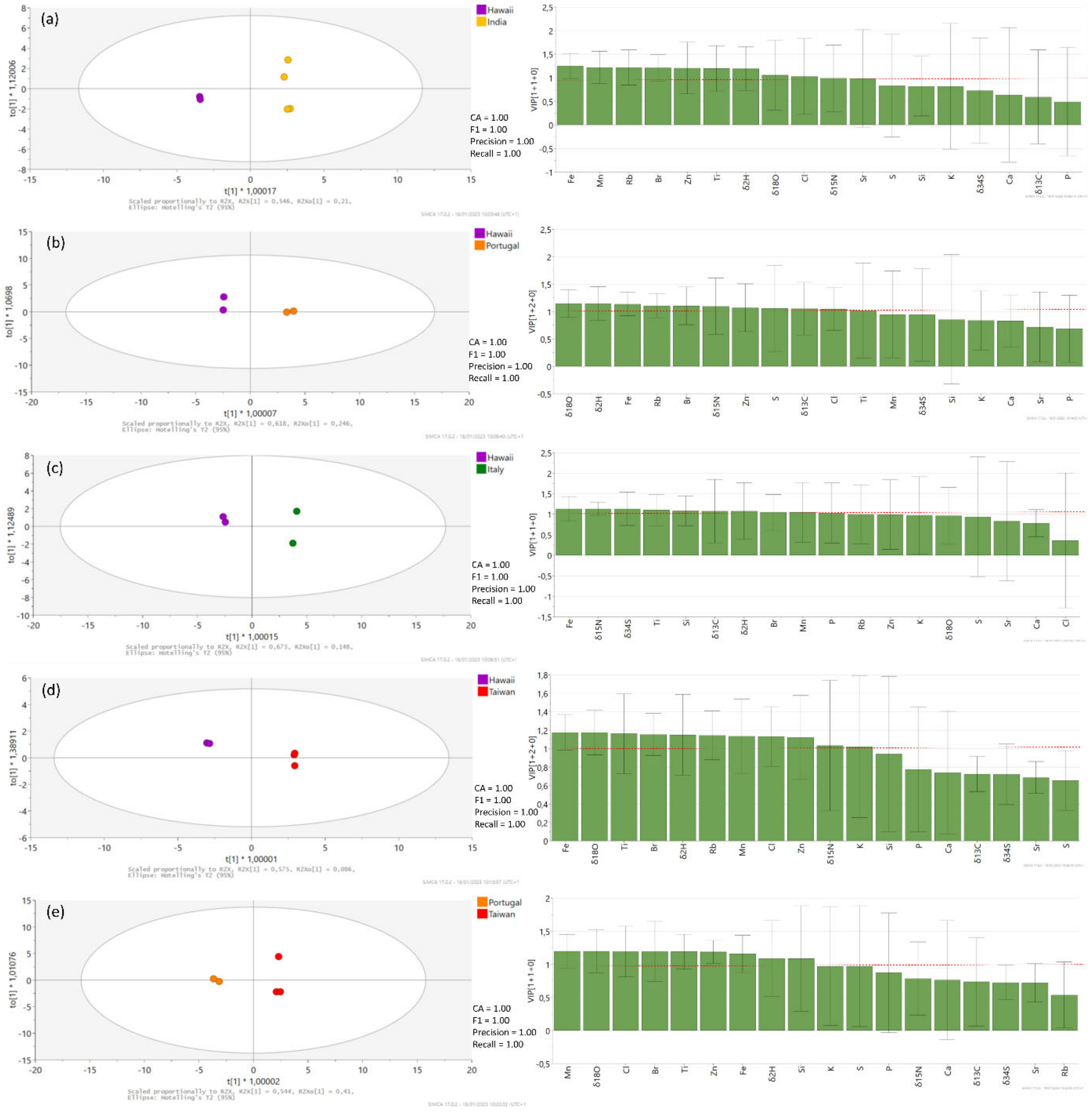

Figure S2: Pairwise comparisons between different declared countries of origin of *Spirulina* products: OPLS-DA score plots and VIP values, derived from all isotopic and elemental composition data. The ellipse on the score plot represents the 95% confidence interval. Red dotted line indicates a criteria for identification of the variables, important for the developed model. Separation of Hawaii from India (a), Portugal (b), Italy (c), Taiwan (d) and separation of Portugal and Taiwan (e).
